# CBSNet: An Effective Method for Potato Leaf Disease Classification

**DOI:** 10.3390/plants14050632

**Published:** 2025-02-20

**Authors:** Yongdong Chen, Wenfu Liu

**Affiliations:** 1 Yuanpei College, Shaoxing University, Shaoxing 312010, China; 2 Hunan Institute of Transportation Engineering, Hengyang 421219, China; 20212894@csuft.edu.cn

**Keywords:** CRMC, STA, BLA, disease detection

## Abstract

As potato is an important crop, potato disease detection and classification are of key significance in guaranteeing food security and enhancing agricultural production efficiency. Aiming at the problems of tiny spots, blurred disease edges, and susceptibility to noise interference during image acquisition and transmission in potato leaf diseases, we propose a CBSNet-based potato disease recognition method. Firstly, a convolution module called Channel Reconstruction Multi-Scale Convolution (CRMC) is designed to extract the upper and lower features by separating the channel features and applying a more optimized convolution to the upper and lower features, followed by a multi-scale convolution operation to capture the key changes more effectively. Secondly, a new attention mechanism, Spatial Triple Attention (STA), is developed, which first reconstructs the spatial dimensions of the input feature maps, then inputs the reconstructed three types of features into each of the three branches and carries out targeted processing according to the importance of the features, thereby improving the model performance. In addition, the Bat–Lion Algorithm (BLA) is introduced, which combines the Lion algorithm and the bat optimization algorithm and makes the optimization process more adaptive by using the bat algorithm to adjust the gradient direction during the updating process of the Lion algorithm. The BLA not only boosts the model’s ability to recognize potato disease features but also ensures training stability and enhances the model’s robustness in handling noisy images. Experimental results showed that CBSNet achieved an average Accuracy of 92.04% and a Precision of 91.58% on the self-built dataset. It effectively extracts subtle spots and blurry edges of potato leaf diseases, providing strong technical support for disease prevention and control in large-scale potato farming.

## 1. Introduction

Potatoes rank as the third-largest food crop globally, cultivated in 158 countries and serving as the primary food source for over 1.3 billion people [1]. However, the rising prevalence of potato plant diseases threatens global production systems [2], particularly as traditional visual inspection methods prove inefficient for large-scale farming. Manual assessments struggle to promptly evaluate disease severity across different regions, creating an urgent need for automated solutions to enable early intervention [3]. Such technological advancements could optimize pesticide usage while maintaining yield stability.

Recent progress in computer vision has driven innovations in crop disease recognition. Early approaches relied on image processing [4] and machine learning [5], requiring labor-intensive manual feature extraction that demanded domain expertise. These methods faced inherent limitations in capturing high-level features and intricate patterns, especially for small or scattered lesions where classifier accuracy significantly deteriorated.

The emergence of deep learning addressed these constraints through automated feature learning. Tang et al. [6] developed ResiAdvNet using asymptotic residuals and adversarial training, achieving a 0.9225 F1 score for potato pest recognition, though its parameter-heavy architecture impedes practical deployment. Reis et al. [7] combined Deep Separable Convolution with Transformer-based attention mechanisms, attaining 99.33% accuracy but showing vulnerability in fuzzy boundary identification due to their majority voting strategy. Shwetha et al. [8] demonstrated 97% training accuracy with LeafSpotNet for jasmine leaf spots, yet conventional CNN frameworks like theirs prioritize macro-textures over microscopic features critical for early disease detection.

Complementary techniques further attempt to resolve computational challenges. Long et al. [9] employed the EXG algorithm in UAV-based systems to extract regions of interest through multi-stage segmentation, reducing processing redundancy. However, like their deep learning counterparts, these methods struggle with real-world noise conditions where subtle features—such as submillimeter spots or blurred edges—are frequently obscured. This persistent limitation stems from inconsistent feature extraction across decision modules, creating a critical bottleneck for field applications.

**Figure 1:**
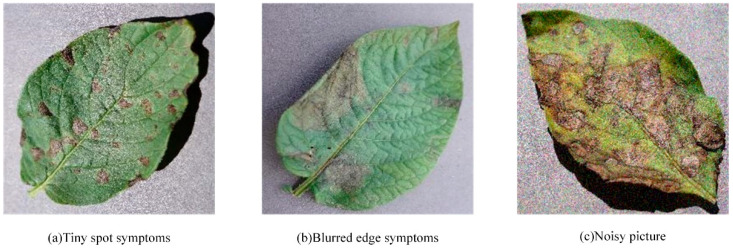
Three challenges in potato disease identification.

Although existing studies have provided an important reference for the agricultural field in potato disease recognition, there are still some limitations in data representation. Most of these studies relied on public datasets and a small number of collected samples, which were usually noise-free and limited in number. This data distribution characteristic makes a model substantially less accurate when dealing with images with noise in practical applications. To address this problem, this paper mainly faces the following challenges: (1) the characteristics of potato leaf diseases are highly similar among species, and some disease symptoms are relatively subtle, so it is easy for the neural network to be interfered with by small and densely distributed spots, which increases the difficulty of feature extraction; (2) the actual acquisition environment is complex and variable, which leads to the blurring of the edge features of the disease region and reduces the recognition accuracy of the model; and (3) images are susceptible to noise during acquisition and transmission, making the model less accurate. The acquisition and transmission process is susceptible to a variety of noise interferences such as sensor noise from cameras, environmental disturbances such as specular reflections from leaves, and motion blurring caused by wind, which can further blur the image features, thus negatively affecting the robustness and accuracy of disease identification. Figure 1 shows these three challenges.

To address the aforementioned challenges, several studies have proposed innovative solutions. Tang et al. [10] introduced a PLPNet-based approach that employs perceptual adaptive convolution to emphasize micro-disease locations on tomato leaves, thereby enhancing the network’s capability to localize spot diseases. Similarly, Nishad et al. [11] utilized the K-means clustering algorithm in combination with VGG16, achieving a 97% accuracy rate on the PlantVillage dataset. This method effectively segregates leaf regions from the background, directing the model’s focus towards the diseased areas where spots appear.

To mitigate the issue of diminished recognition accuracy caused by blurred disease edges, Dai et al. [12] developed the Transformer-based AISOA-SSformer model. This model combines spatial and channel-wise reconstructive processing with a sparrow algorithm that expands the search space, enhancing the model’s ability to detect fuzzy boundary features while maintaining training stability. Additionally, Arshad et al. [13] introduced PLDPNet, an end-to-end deep learning framework for detecting potato leaf diseases. PLDPNet leverages deep features from VGG19 and Inception-V3, utilizing VGG19′s strength in capturing fine-grained local features and Inception-V3′s ability to extract multi-scale textures. The integration of the sparrow algorithm further strengthens the model’s capability to identify indistinct boundaries, ensuring both stability during training and overall robustness.

To address the problem of low model robustness brought by noisy images, Zhao et al. [14] proposed DoubleGAN, a dual-generation adversarial network, balancing the dataset by generating unhealthy plant leaf images, in which the first stage of WGAN generates the initial image by pre-training the model with both healthy and unhealthy leaves to improve the base quality of the generated results and the second stage of SRGAN focuses on improving resolution and suppressing noise. Hou et al. [15] proposed to accurately segment leaf regions by an improved graph cut algorithm and extract features by combining Lab* color space and a Local Binary Pattern (LBP). Finally, an SVM [16] classifier was used to achieve 97.4% and 91.0% accuracy in the classification of the disease type and degree of infection, respectively, combining Otsu threshold segmentation and color statistical thresholding to extract foregrounds and backgrounds, which made the network highly noise-resistant.

The contributions of this paper are as follows:

1. In order for the image classification network to acquire rich potato leaf disease features, we constructed a classification dataset containing early-blight, late-blight, and healthy leaves.
2. We propose a convolution method called Channel Reconstruction Multi-Scale Convolution (CRMC). This method ensures the stability of classification of tiny diseases by separating the channel layer features, extracting the upper and lower layer features using a more optimal convolution and subsequently responding to key variations more efficiently through a multi-scale convolution operation.
3. A new attention mechanism, Spatial Triple Attention (STA), is proposed, whereby the spatial dimensions of the input feature map are reconstructed and then the reconstructed three types of features are inputted into the three branches, and more targeted operations are carried out according to the importance of the features, so as to achieve the accurate classification of fuzzy edge elements.
4. A new optimization algorithm, the Bat–Lion Algorithm (BLA), is proposed. This algorithm combines the Lion algorithm and the bat optimization algorithm to make the optimization process more adaptive by using the bat algorithm to update the direction of the gradient during the Lion update process. Its dynamic frequency and loudness adjustment allows the optimizer to have a larger step size in the exploration phase and gradually reduce the step size in the convergence phase, which helps avoid overfitting and find the optimal solution more finely.
5. The CBSNet proposed in this paper obtained an average accuracy of 92.04% and Precision of 91.58% on a self-constructed potato disease dataset. The experimental results showed that the method can effectively classify potato leaf diseases and provide a reference for disease prevention in large-scale potato production.

## 2. Materials and Methods

### 2.1. Data Acquisition

Effective recognition and classification of potato diseases depend on the optimization and refinement of algorithms, performance evaluation, and the availability of comprehensive datasets. To ensure ample training data for image-based disease identification, we developed a potato plant disease dataset derived from PlantVillage. To maintain the quality of input images, all samples were categorized into three classes: Early Blight, Healthy, and Late Blight. The dataset comprises a total of 2450 images, which were split into 1960 images for training and 490 images for validation, following an 80:20 ratio. All images are stored in jpg format, as detailed in Table 1.

### 2.2. Method

#### 2.2.1. Channel Reconstruction Multi-Scale Convolution (CRMC)

**Figure 2:**
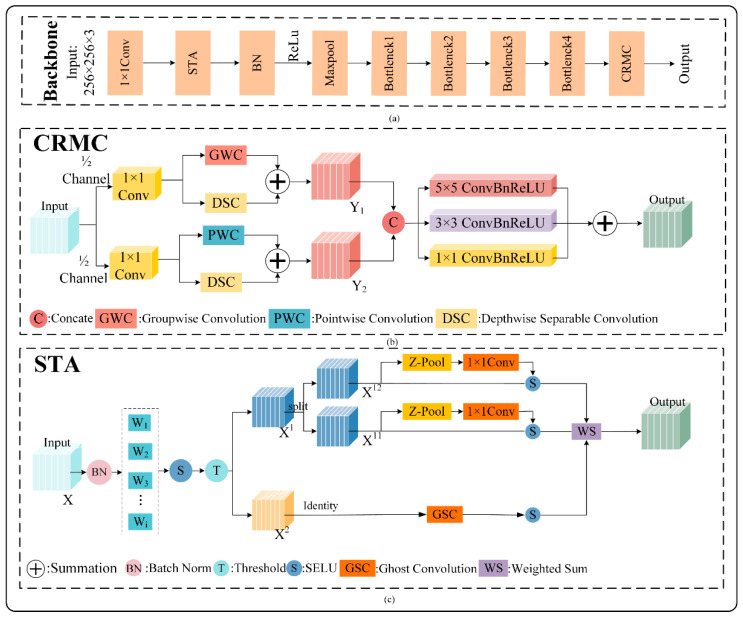
Structure diagram of CBSNet. (**a**) represents the overall network structure model of CBSNet, (**b**) represents the CRMC structure, and (**c**) represents the STA structure.

In potato pest classification, larger datasets often increase the demand for computational resources and can impact a model’s generalization ability. While the initial convolution operation of the ResNeXt-50 model can capture some microscopic speckled disease features in maize pest data, the use of a single 7 × 7 convolution kernel limits effective feature extraction. This constraint hinders the model’s ability to process numerous small speckle features and restricts its ability to analyze deeper image features. To tackle the challenge of reduced generalization caused by small speckle features in the input data, this paper introduces a new method called Channel Reconstruction Multi-scale Convolution (CRMC). CRMC is designed to process image features at deeper levels, effectively capturing tiny spot details and improving the model’s generalization capability. The architecture of CRMC is shown in Figure 2b.

Multi-scale convolution enhances the detail information of microscopic lesions on potato plants by applying various convolution operations to the input feature maps. This design was inspired by the Multi-Scale Convolution Module (MCM) proposed by Deng et al. [17], which demonstrated the effectiveness of multi-scale convolution in extracting fine spot disease features. Compared to single-scale convolution, multi-scale convolution offers superior feature expressiveness with only a moderate increase in computational overhead. However, directly concatenating multi-scale features can result in semantic inconsistencies between different scales, diminishing the effectiveness of the final feature fusion. To overcome this challenge, Channel Reconstruction Multi-scale Convolution (CRMC) module is introduced prior to the multi-scale convolution operation. CRMC performs hierarchical processing and semantic adjustment of the input feature maps through channel segmentation and reconstruction. This process generates more consistent and distinguishable channel features, facilitating better detection of tiny lesions. Consequently, CRMC enhances both the accuracy and robustness of feature representation, making it well suited for identifying subtle disease manifestations in potato plants.

The operation flow of Channel Reconstruction Multi-Scale Convolution (CRMC) is as follows: firstly, the input feature map is divided into two parts according to the channel dimension: the upper channel and the lower channel. The upper channel is responsible for capturing macroscopic features while the lower channel focuses on microscopic features, with a division ratio of 1:1. Then, the upper channel is dimensionality reduced, and the number of channels is compressed by 1 × 1 convolution to reduce the computational burden and strengthen the key features. Subsequently, the upper channel extracts inter-group features by group convolution (GWC) and further extracts intra-channel features by depth separable convolution (DSC) [18] and finally sums and fuses the outputs of both. At the same time, the lower-layer channel also undergoes dimensionality reduction and extends the channel dimensions by point-by-point convolution (PWC) while applying depth separable convolution to obtain fine-grained features, then the results of the two are spliced to complete the construction of the lower-layer features. Then, the features from the upper and lower channel processing are re-spliced to complete the channel reconstruction. Finally, multi-scale convolution is applied to the reconstructed feature maps, including 1 × 1, 3 × 3, and 5 × 5 convolution operations, to capture different sizes of receptive fields and to enhance the feature expression capability by combining batch normalization and activation functions. Ultimately, the results of multi-scale convolution are spliced in the channel dimension to achieve a rich and consistent feature representation and obtain the final feature map.

Specifically, we designed a structure combining channel reconstruction and multi-scale convolution to cope with the problem of high interference in neural networks in the classification of tiny spots on potato leaves. First, we split the input feature maps along the channel dimension into upper and lower channels, with each focusing on macro and micro features. The upper channel extracts inter-group and intra-channel features using group convolution and depth-separable convolution while the lower channel employs point-by-point convolution and depth-separable convolution to enhance the representation of fine-grained details. Next, the reconstructed channel features are passed through the multi-scale convolution module, where different convolution kernels (e.g., 1 × 1, 3 × 3, and 5 × 5) capture varied feature representations. The quality of these features is further enhanced through normalization and activation functions. Finally, the fused feature maps are fed into the classification network, which leverages both the reconstructed features and multi-scale information to boost classification accuracy and generalization ability. This approach, combining channel reconstruction with multi-scale convolution, effectively captures the critical features of tiny lesions while optimizing feature semantic consistency, offering a robust solution for detecting potato leaf diseases in complex environments. The experimental results are provided in Section 3.3.1.

#### 2.2.2. Spatial Triple Attention (STA)

To address the issue of blurred disease edges in potato pest leaves, we employ an attention mechanism to enhance the model’s ability to extract features from blurred disease areas. The attention mechanism helps the model focus on the critical regions of the input data, improving its sensitivity to disease edges, while effectively filtering out noisy background elements. This reduction in background interference boosts the model’s performance in complex environments, increasing its recognition accuracy and robustness.

In deep learning, the attention mechanism enhances the network’s ability to extract features by guiding the model to focus on important target areas. Attention mechanisms are widely used in neural networks and have been shown to significantly improve the performance of visual models [19,20,21]. For example, the Squeeze-and-Excitation (SE) module [22] adds attention to the channel dimension, adaptively learning the weights for each channel and assigning varying importance to each feature. This not only boosts network performance [23] but also improves its robustness. The Triplet Attention (TA) mechanism [24] takes this further by using a three-branch structure to capture cross-dimensional interactions, enabling the computation of attention weights that capture more contextual and long-range dependencies. Building on these approaches, we propose a new attention mechanism, Spatial Triple Attention (STA), which enhances the classification of blurred edge regions. STA works by first reconstructing the spatial dimensions of the input feature map and then passing the reconstructed features through three separate branches for more refined processing. The structure of STA is illustrated below, with the following specific operations.

Firstly, we normalize the input potato disease feature maps by batch normalization, aiming to standardize the input feature maps and reduce the bias in the training process. Then we use the SELU activation function to generate a weight value W in the range of (0,1), then use the gating mechanism to split the feature map, the gating mechanism according to the threshold we set to divide the weights into two parts, one part of the potato disease feature map of the high-informativeness features, and one part of the potato disease feature map of the low-informativeness features, and we are in the subsequent operations. The high-informativeness features are focused on, and the low-informativeness parts of the features are multiplied by the scaling factor, which reduces, but does not completely erase, their role in network training, and the formula for the above operation is as follows:

(1) $$X=\frac{X-\mu }{\sqrt{\sigma^{2}}+\varepsilon }$$

(2) $$weights=SELU\left(X\right)$$

(3) $$SELU=\left\{\begin{array}{l} \lambda \cdot X,\;\;\;\;\;\;\;\;\;\;\;\;\;\;\;\;\;\;\;if\;X>0\\ \lambda \cdot \alpha \cdot \left(e^{X}-1\right),\;if\;X\leq 0 \end{array}\right.$$

(4) $$X_{1}=\left\{X_{i,j}|weights\left(i,j\right)\geq threshold\right\}$$

(5) $$X_{2}=alpha\times \left\{X_{i,j}|weights\left(i,j\right)<threshold\right\}$$

**Figure 3:**
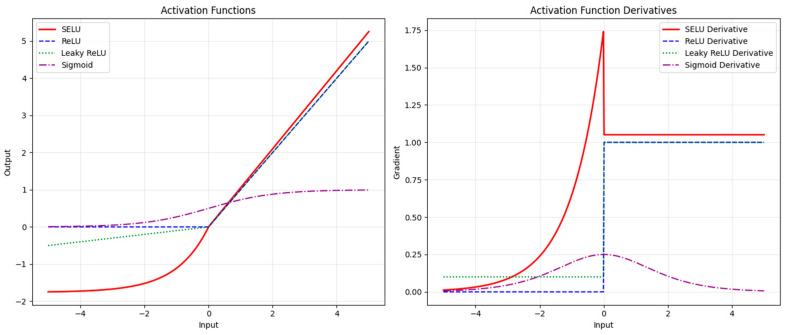
Comparison of SELU function with other common activation functions.

A comparison of the output of the SELU activation function with the rest of the common activation functions is shown in Figure 3, where SELU exhibits superior performance in deep neural networks through three key mechanisms. During training, its self-normalization property essentially scales the activations towards zero mean and unit variance, thus reducing the reliance on explicit normalization layers. Second, the non-zero gradient in the negative region mitigates the “dead neuron” problem prevalent in ReLU-based architectures while maintaining gradient stability. Third, the unconstrained positive output and smooth saturated negative response of SELU achieve a wider dynamic range than the equivariant function, effectively mitigating the problem of gradient vanishing.

We then process the high-information-content features by first dividing them into two parts according to their numbers of channels, denoted as $X_{11}$ and $X_{12}$. In order to allow the attention mechanism to focus on more distinct sub-features, we first subject $X_{11}$ and $X_{12}$ to the Z-POOL [25] operation, which channel-aggregates the feature maps, which allows it to retain a rich representation of the actual tensor while shrinking its depth in order to make the further computations lightweight. Next, the spatial attention map is generated by a convolution operation with a convolution kernel size of 1. Finally, the spatial attention map is controlled by the SELU [26] activation function, which controls the value of each pixel of the spatial attention map to be in the range of [0, 1]. For the low-informative feature $X_{2}$, we use the Phantom Convolution (Ghost Convolution) method [27], which reduces the complexity by generating fewer feature maps and yet maintains a better performance, and finally, through the SELU activation function, it is weighted with the outputs of the informative two branches. In this way, the high-value region of the spatial attention map enhances the features at the corresponding positions in the input feature map and the low-value region reduces the influence of the corresponding features (e.g., the fuzzy part). The specific formula is as follows:

(6) $$Z-Pool\left(X\right)=\left[MaxPool_{0d}\left(X\right),AvgPool_{0d}\left(X\right)\right]$$

(7) $$X_{out}=\frac{1}{3}\left(X_{2}+X_{11}+X_{12}\right)$$

This method effectively improves the feature capturing ability of potato leaf diseases by introducing the STA module. By distinguishing high-informativeness and low-informativeness features through the gating mechanism, we are able to focus on features with higher information density, thus optimizing the learning process of the network. For low-information-content features, the Phantom Convolution operation is used to effectively reduce the computational complexity while maintaining the richness of the feature map. The channel aggregation of high information content features by Z-POOL operation and the combination of spatial attention maps enable the model to automatically focus on the key regions, which further enhances the recognition of foreground features. Ultimately, the combination of the SELU activation function and the weighting operation ensures that features in high-informativeness regions are amplified while low-informativeness regions are effectively suppressed. This integration strategy not only improves the classification accuracy of potato leaf diseases but also enhances the robustness and efficiency of the model in the face of fuzzy edges, providing a more accurate solution to the potato disease classification task. Experiments on STA are described in Section 3.3.2.

#### 2.2.3. Bat–Lion Algorithm (BLA)

**Figure 4:**
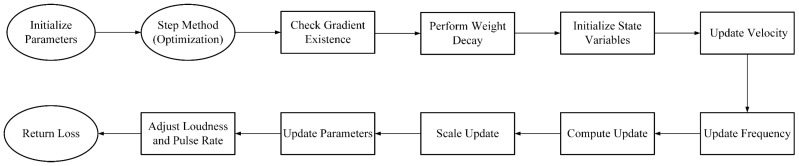
Flowchart of BLA.

To enhance model stability during training and improve classification accuracy, we introduce the Bat–Lion Algorithm (BLA) for parameter optimization. The dynamic adjustments of parameters (such as learning rate and momentum coefficient) during training play a crucial role in the model’s robustness and accuracy. However, traditional parameter optimization methods face a dual challenge in coping with noise-laden potato disease images [28]: first, the local noise of leaf spots distorts the gradient direction, leading to oscillations in parameter updating caused by symbol updating rules incorrectly amplifying interfering signals; and second, the interclass differences in leaf diseases are often manifested in subtle texture variations, which requires the optimizer to have the ability to dynamically adjust the exploration accuracy. To this end, we propose the Bat-Lion algorithm, based on Lion [29], which encodes the intelligent search mechanism of bat echolocation [30] into a gradient optimisation framework. Specifically, the dynamic frequency modulation mimics a bat’s behavior of regulating ultrasonic frequency to search for prey, and through adaptive oscillation of the frequency parameter (controlled by pulse rate), we quickly locate the common features of diseased patches with high-frequency small-amplitude updating in the initial stage and turn to low-frequency large jumps to escape from the locally optimal region of healthy leaves in the later stage. The loudness attenuation mechanism is used to emulate the strategy of bats of reducing the intensity of their calls when approaching a target, and a smooth transition from coarse-grained global search to fine-grained local tuning is realized through exponential attenuation to ensure that the model accurately distinguishes confusing diseases at the late stage of convergence. The impulse perturbation strategy transforms the characteristic of bats randomly emitting impulse waves into a stochastic scaling factor of gradient amplitude, which enhances the robustness to local disturbances in diseased foliage images through controllable noise injection while preserving the directionality of the original symbol update. This strategy enables BLA to combine the high efficiency of gradient optimization with the noise tolerance of evolutionary algorithms, providing a more robust training method for deep learning models. The flowchart of the BLA is shown in Figure 4.

At the beginning of training, BLA first initializes the model parameters and their exponential moving average of gradient and momentum. The optimizer then verifies the hyperparameters such as the learning rate, momentum coefficients, and sliding average coefficients of the gradient squares, the reasonableness of which directly affects the stability of the update path and the convergence efficiency. To suppress overfitting, the algorithm prioritizes the execution of the weight decay operation: a penalty term proportional to the squared weight paradigm is introduced in the loss function via L2 regularization [31]. At its core, it applies a continuous contraction pressure on the larger weights, forcing the network parameters to converge to a distribution with a smaller absolute value. This mechanism not only alleviates the model’s oversensitivity to noise or outliers in the training data but also enhances parameter smoothness by constraining the degrees of freedom of the weights, thus improving the model’s generalization ability on unknown diseased leaf data. Unlike the sparsifying property of L1 regularization, the quadratic property of L2 penalty retains the continuous minimizability of the weights, which is more suitable for dealing with high correlations among leaf features, such as the symbiotic relationship between lesion texture and color distribution, and guides the weights to asymptotically go to zero while maintaining the collaborative information of the features to avoid destroying the convolutional layer’s ability to locally sense the disease signs due to strict sparsification.

Subsequently, the optimizer updates the exponential moving average of the gradient by combining the current gradient information through a momentum update operation. This operation accelerates the learning process by exploiting the inertia effect of the historical gradient direction and suppresses parameter oscillations due to local differences in the leaf image, allowing the update direction to align more stably with the discriminative feature space. The introduction of the momentum mechanism allows the optimizer to continuously advance towards the optimal region along the “inertial trajectory” of the loss function, which significantly reduces the risk of falling into local minima, especially when dealing with flat optimization surfaces, which are widely found in diseased leaf data. Thereafter, the algorithm fuses the current and historical gradient information and performs a momentum decay update. This mechanism smoothes out short-term fluctuations in the gradient direction, such as the noise gradient induced by the transportation process in a single batch of images, by dynamically adjusting the momentum weights and gradually reduces the update step size as the model approaches the optimal solution to avoid crossing the optimal boundary of disease classification due to a large step size. The synergy of momentum decay and bio-inspired frequency modulation allows the algorithm to calibrate the classification decision surface with fine step sizes in the later stages of training while retaining the ability to escape from sub-optimal solutions. The specific process is described below:

(8) $$p_{new}=p_{old}\times \left(1-\eta \lambda \right)$$

(9) $$v_{t}=\beta_{1}\times v_{t-1}+\left(1-\beta_{1}\right)\times \nabla L\left(\theta \right)$$

Here, η is the learning rate, λ is the weight decay parameter, $\beta_{1}$ is the momentum coefficient, and ▽L(θ) is the gradient of the loss function with respect to parameter θ.

Then we add the idea of bat optimization algorithm, in which bats explore a wider solution space by constantly adjusting their flight frequency. In BLA, the frequency is dynamically updated and is affected by the pulse rate and the stochastic factor. At each update, the frequency is changed to explore new solution space so as to avoid falling into local optimal solutions. The update formula for frequency is as follows:

(10) $$f_{new}=f_{old}\times \left(1+random\times pr\right)$$

Here, random is a random factor and pr is pulse rate, which is used to adjust the rate of change of frequency. This dynamic frequency update process makes the optimizer more exploratory.

In the bat optimization algorithm, loudness is an indication of the intensity of the sound emitted by the bat. In BLA, loudness controls the magnitude of the gradient update. As training proceeds, loudness gradually decays (i.e., decreases the step size) to help the optimizer make finer adjustments as it converges. The process of loudness decay and symbol-based gradient update is as follows:

(11) $$A_{new}=A_{old}\times 0.99$$

(12) $$\Delta \theta =-\eta \times f\times A\times sign\left(v_{t}\right)$$

BLA adds the dynamic frequency and loudness adjustment mechanism of the bat algorithm to the traditional Lion optimizer, making the optimization process more adaptive. The dynamic frequency and loudness adjustment allows the optimizer to have a larger step size in the exploration phase and gradually reduce the step size in the convergence phase, which helps avoid overfitting and find the optimal solution in a more refined way. BLA improves the stability of the model training, and enhances the accuracy and robustness of the classification of potato diseased leaves. Experiments on BLA are described in Section 3.3.3.

## 3. Results and Analyses

### 3.1. Experimental Environment and Training Details

The main hardware devices used in our experiment were an RTX 3090 GPU and 12 vCPU Intel(R) Xeon(R) Platinum 8255C CPU. While the versions of Python 3.8.10, CUDA 11.3, and CUDNN 8.2.4 did not affect the results of the experiment, they needed to be compatible with the software and hardware. We implemented CBSNet on Pytorch 1.11.0. The specific hardware table is shown in Table 2.

The size of the input images was 256 × 256 pixels, and 2450 images of potato leaf disease were divided into training and test sets in the ratio of 8:2. Before training, data enhancement operations were performed on the dataset, specifically perspective transformation, mirror flip, and contrast adjustment. First of all, in the actual environment, potato leaves are often photographed at different angles due to uneven plant growth or different camera placement. Perspective transformations can simulate these natural variations in perspective and enhance a model’s ability to recognize lesions and edges in the presence of geometric distortions. Secondly, lesions may behave symmetrically or asymmetrically depending on environmental factors (e.g., sunlight exposure). The use of mirror flipping ensures that the model does not overfit the orientation bias in the training data. Finally, field images are often affected by uneven lighting. Adjusting contrast improves robustness to lighting variations and ensures that features can be reliably extracted even under sub-optimal lighting conditions that are common in real-world agricultural scenarios. We used these three data enhancement methods to simulate key variability in real-world potato growing environments while avoiding excessive synthetic distortion that could skew disease patterns. The final result comprised 7350 images. The experiment consisted of 100 training cycles, the learning rate was 0.0001, the optimizer used the BLA, the weight decay coefficient was set to 1e-8, and the momentum was 0.9. The batch_size value was set to 16, considering the performance of the hardware device and the training effect.

### 3.2. Evaluation Indicators

To evaluate the potato diseased leaf classification results, we used Accuracy, Precision, Recall, and F1 score. Accuracy represented the proportion of correctly predicted samples, Precision measured the proportion of predicted diseased leaves that were actually diseased, Recall reflected the proportion of actual diseased leaves correctly identified by the model, and the F1 score was the harmonic mean of Precision and Recall, providing a comprehensive assessment of model performance. The formulas for these metrics were as follows:

$$Precision_{i}=\frac{TP_{i}}{TP_{i}+FP_{i}}$$

$$Recall_{i}=\frac{TP_{i}}{TP_{i}+FN_{i}}$$

$$F1-score_{i}=2\times \frac{precision_{i}\cdot Recall_{i}}{precision_{i}+Recall_{i}}$$

$$A\mathrm{cc}=\frac{1}{n_{c1}}\sum_{i=1}^{n_{c1}}\frac{n_{ij}}{n_{i}}$$

Here, TP, FP, and FN denote true positives, false positives, and false negatives, respectively. The parameter i represents the category index and n represents the total number of categories. The variable denotes the number of correctly predicted initial categories, where j represents the prediction result and i denotes the category index. The variable then denotes the number of initial categories that have been accurately predicted.

### 3.3. Experimental Results and Analyses

#### 3.3.1. Validity of CRMC

In this paper, we use CRMC instead of the original 1 × 1 convolution of the output convolution layer to process the output feature maps, and we compare the performance of 1 × 1 Convolution, Ghost Convolution [27], DSC [18], Atrous Convolution [32], and Involution [33] to determine the output convolution layer’s form, and the results are shown in Table 3.

The experimental results showed that after trying to replace the convolution, all the four convolutions except DSC had improved due to the fact that DSC first processes each channel through deep convolution and then merges these channels through point-by-point convolution. This may have led to the ineffective fusion of features between different dimensions at the beginning of the network, resulting in a less smooth transfer of information than with standard convolutional layers. Ghost Convolution saves computational resources but also leads to a lack of sufficient expressive power. Whereas Atrous Convolution extends the sensory field, it does not increase the fine-grained capture of local features that are crucial for subsequent layers. Involution operations typically compute based on the local information of the input feature map, but they do not capture global information at an early stage the way standard convolution does. Our proposed CRMC performs a multi-scale reconstruction operation on features in the channel dimension, so it works best in potato leaf disease classification.

#### 3.3.2. Validity of STA

In this paper, we use STA after 1 × 1 convolution to enhance the feature extraction ability of the model, and in order to verify its effectiveness, we choose to compare it with CBAM [34], Coordinate Attention (CA) [23], Criss-cross Attention (CCA) [35], and TA [24]. The experimental results are shown in Table 4.

The experimental results demonstrated that, except for CBAM, all other attention mechanisms can filter out interference information and improve segmentation accuracy to some extent, although the effect is not significant. This can be attributed to the fact that CBAM places excessive emphasis on certain local regions while neglecting global features, resulting in poorer classification performance. While CA considers feature information in two dimensions, it still struggles to capture contextual information effectively. On the other hand, TA focuses on applying attention across different dimensions but fails to integrate this information effectively, leading to sub-optimal performance. In contrast, STA proves to be more effective for feature extraction in the context of potato leaf disease classification.

#### 3.3.3. Effectiveness of the BLA

In this paper, we incorporate a bat optimization algorithm based on Lion to improve the model’s ability and robustness to classify noisy potato leaf diseases. We experimented with several state-of-the-art optimizer algorithms (Adam [36], Lion [29], Radam [37], RMSprop [38], Grey Wolf Optimizer [39], and Sparrow Search Algorithm [40]) in the same experimental configuration, and the and experimental results are shown in Table 5.

In this study, we conducted systematic comparative experiments on the performance of seven optimizers for the potato disease classification task. The experimental results showed that the BLA, which was improved based on the bio-heuristic algorithm, had the best overall performance, ranking first in terms of Accuracy (87.45%), Precision (87.76%), and F1 score (83.36%), while the Recall (85.37%) was also at a sub-optimal level. Among the traditional optimizers, Lion and Radam showed strong competitiveness with 86.96% and 86.88% accuracy, respectively, but there was a performance gap of about 0.5% over the BLA in terms of the F1 score. Although the Gray Wolf Optimizer (GWO) achieved the highest single-indicator recall (86.94%), there was an obvious imbalance between its Recall and F1 score. The experimental results verified the effectiveness of the hybrid optimization strategy, and the BLA significantly improved the recall rate (0.6% improvement over the sub-optimal method) while maintaining high precision by fusing gradient information with the bat population intelligence mechanism, a balance that makes it more suitable for the disease recognition needs of category imbalance in real agricultural scenarios.

#### 3.3.4. Ablation Experiments

To validate the effectiveness of the proposed method, we conducted ablation experiments on CBSNet, with the results being presented in Table 6. Using a control variable approach, we sequentially integrated the CRMC, STA, and BLA modules into the network and performed eight sets of experiments combining these components. The results demonstrated that STA significantly enhances classification performance. Specifically, adding STA alone improved Accuracy by 2.88% and Precision by 4.30%, indicating its ability to accurately extract features from the input data and enhance classification accuracy. Furthermore, when CRMC and the BLA were incorporated, the improvements in Accuracy (2.40%, 2.76%) and Precision (3.06%, 3.30%) were smaller but still noteworthy. These findings confirm that CRMC, STA, and the BLA are all effective in improving the performance of the CBSNet model for potato leaf disease classification.

#### 3.3.5. Comparative Experiments with State-of-the-Art Methods

**Figure 5:**
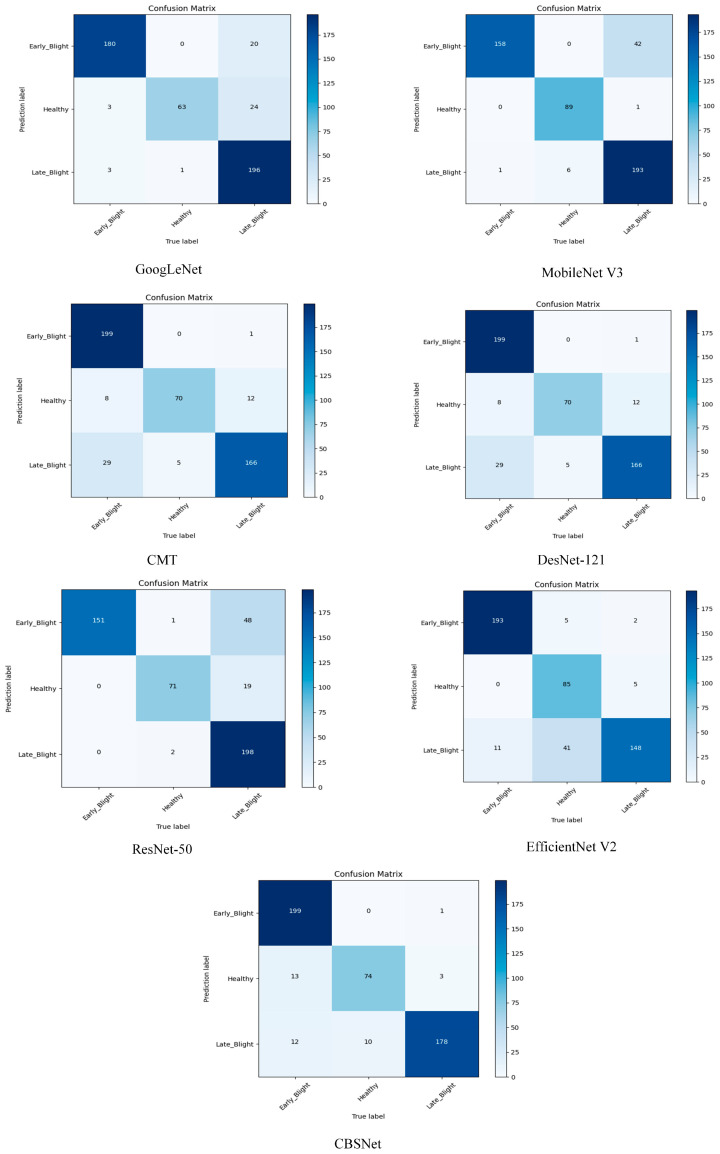
Confusion matrix comparison chart.

To further analyze the performance of CBSNet, we conducted comparative experiments with traditional and current state-of-the-art target detection methods on the same test environment and test set. The experimental results and confusion matrix are shown in Table 7 and Figure 5.

In order to further analyze the performance advantages of CBSNet, we compared it with both traditional convolutional neural network architectures as well as some advanced networks proposed in recent years. We found that among the classical convolutional neural networks, GoogLeNet uses multi-scale convolution, which not only reduces the computational effort of the model but also improves its ability to fuse fine- and coarse-grained information. However, its multiple branches and containing too many hyperparameters have high requirements for hardware and tuning, which makes it difficult for it to perform its best classification ability. ResNet-50 effectively solves the problems of gradient disappearance and explosion in traditional networks through the residual structure, and it has strong generalization ability. However, it relies more on the network to learn the features of each residual block correctly, and if the learned features are highly interfering, it will be difficult for it to realize the potential brought by the depth. The core idea of DensNet-121 is the dense connectivity, which allows the later convolutional layers to access the features of all the previous convolutional layers and thus improves the diversity of the feature expression. However, the design is applied to the overall network, and it is difficult for it to achieve higher efficiency with lower computation. CMT tries to combine the local perception ability of convolutional neural networks with the global modeling ability brought by the self-attention mechanism in Transformer, which can capture more global contextual information. However, its tuning is difficult and facing higher resolution images will make the computational complexity grow exponentially.

For a lightweight network structure, EfficientNet V2 has a progressive learning strategy, which can speed up the overall training; however, this ‘phase’ and ‘module mixing’ approach makes the overall structure of the network more complex, and the switching rules between different phases need to be carefully designed. MobileNetV3 combines separable convolution and SE attention mechanism, which reduces the computation volume and improves the model accuracy. However, since part of the model structure is automatically searched, if further changes or the fine-tuning of the network structure are needed, it is often necessary to conduct a new search or a large number of experiments, which leads to the difficulty of manual parameter tuning.

In contrast, CBSNet outperforms recent networks in terms of overall performance. Channel Reconstruction Multi-Scale Convolution is able to better separate the channel layer features and perform multi-scale feature extraction, enabling the network to achieve a more effective response to micro-diseases. Then Spatial Triple Attention is used to perform more targeted operations based on the importance of the features, and finally, the network training is optimized by the BLA to improve the network robustness. Finally, the experimental accuracy reached 92.04%, which indicates that our proposed CBSNet can improve the accuracy of potato diseased leaf classification.

## 4. Discussion

In addition, in order to verify the model’s ability to recognize other types of diseases, we downloaded the public datasets Plant-Disease and Crop Disease Image Classification Dataset from Kaggle and conducted experiments. Plant-Disease includes 38 categories of diseases affecting the more common plants such as apple, cherry, grape, etc. We selected the apple category for generalization experiments, and the Crop Disease Image Classification Dataset (CDICD) includes five different categories—Cassava Bacterial Blight, Cassava Brown Streak Disease, Cassava Green Mottle Mottle Disease, and Cassava Mosaic Disease. These two datasets have a richer variety of classifications, which can show the effect of CBSNet more intuitively. The experimental results are shown in Table 8, with a mean accuracy of 97.41%.

Compared to other models, CBSNet proved to be the most effective for potato leaf disease classification. We attribute this superior performance to several key factors. First, to highlight the subtle disease features in potato leaves, CBSNet employs separated feature operations and multi-scale convolution, which enhances the visibility of these tiny disease-related features. To capture fuzzy edge features accurately, the model incorporates a triple-targeting operation, which enables the precise classification of blurred boundary elements. Additionally, to address model overfitting and stabilize training, we apply the BLA to smooth the loss curve, further enhancing the model’s robustness. As demonstrated by the experimental results, these strategies significantly improve classification accuracy for potato diseases.

CBSNet has demonstrated strong performance in classifying potato leaf diseases and shows promising results for identifying various other plant leaf types. The model excels in accurately detecting two common potato diseases thanks to its robust feature extraction capabilities. However, due to the wide variety of potato diseases and the complex nature of leaf features, it is not yet able to identify all disease types comprehensively. In particular, its accuracy could be improved for rare diseases or those with similar characteristics. Additionally, the current method is focused mainly on disease detection at the image acquisition stage and does not yet support the large-scale monitoring of the entire potato cultivation process or track disease dynamics.

Looking ahead, to enhance the practicality and scalability of this method, we propose integrating additional technologies such as the Agricultural Internet of Things (IoT), big data analytics, and UAV-based remote sensing for real-time monitoring and prediction. This integration could facilitate the monitoring of environmental conditions, pest and disease outbreaks, and their spread, leading to improved agricultural management. Such advancements would not only promote the modernization and precision management of the agricultural industry but also foster the development of agricultural intelligence, ultimately improving potato yield and quality while safeguarding farmers’ income and food security.

## 5. Conclusions

Aiming at the problems of training stability caused by tiny disease features, fuzzy edge textures, and image noise in the potato leaf disease classification task, we propose a network called CBSNet, which achieves better results in potato leaf disease classification.

a. Ablation experiments have demonstrated that CRMC, STA, and the BLA significantly enhance the classification of potato leaf diseases, resulting in accuracy improvements of 2.40%, 2.88%, and 2.76%, respectively. Additionally, under the same experimental conditions, CBSNet outperforms ResNeXt-50, achieving a 7.80% increase in Precision.
b. Comparative experiments conducted on our collected potato leaf disease dataset showed that our Accuracy was 92.04%, Precision was 91.58%, Recall was 90.24%, and F1 score was 90.71% compared to CMT, which had been the best performer in the network in recent years. The experimental results showed that the method had obvious advantages in extracting the features of tiny spots and fuzzy edges in potato leaf diseases.
c. The CBSNet model performed excellently in the generalization experiments on two currently dominant public datasets, Plant-Disease and CDICD. The results of the model’s generalization experiments underline its potential for practical applications, especially in important areas such as plant disease classification.

In this paper, we present an innovative potato leaf disease classification network, CBSNet. Ablation experimental results showed that the CRMC, STA, and BLA modules are effective for potato leaf disease classification, improving the accuracy by 2.40%, 2.88%, and 2.76%, respectively. Under the same experimental setup, CBSNet improved by 7.80% in Precision metrics compared to the ResNext-50 model. In the comparison experiments conducted on our collected potato leaf disease dataset, CBSNet achieved 92.04% Accuracy, 91.58% Precision, 90.24% Recall, and 90.71% F1 score, which significantly outperformed the CMT model, which had been the best performing model in the network in recent years, and verified its high accuracy in potato leaf disease classification. The experimental results showed that CBSNet has obvious advantages in extracting tiny lesions and fuzzy edge features in potato leaves. In addition, the CBSNet model also performed excellently in generalization experiments conducted on two current mainstream public datasets, Plant-Disease and CDICD, further emphasizing its wide potential for practical applications, especially in important areas such as plant disease classification. These properties make CBSNet a powerful tool to support the monitoring and management of agricultural pests and diseases, providing accurate and reliable technical support for improving potato yield and quality.

However, there is still room for exploration in various aspects of its future development and application. First, the cross-crop adaptability of the model needs to be further verified. For example, in the task of disease classification in different crops such as wheat and tomato, the differences in leaf morphology and disease characteristics may pose a new challenge to the generalization ability of the existing modules. In addition, the impact of actual complex environmental factors on model robustness has not been systematically evaluated, and the applicability of the model can be improved by fusing multimodal data (e.g., near-infrared and text labeling) in the future. Second, the current model relies on high-quality data while the acquisition of disease images in real agricultural scenarios is often limited by the cost of equipment and field conditions. Finally, we hope to build a real-time monitoring platform by combining the feedback from practical applications in the future, and push CBSNet from the laboratory to the field applications, so as to realize a wider socio-economic value in the smart agriculture ecology.

## Figures and Tables

**Table 1:** Data set composition.

Category	Example	Characteristics	Proportion
Early Blight	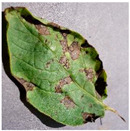	Concentric ring brown lesions on the leaves, usually accompanied by early leaf shedding, mainly caused by fungi, mostly in high-temperature and high-humidity environment	40.82%
Healthy	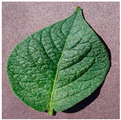	Healthy potato leaves are usually bright-green; uniform in texture; free of plaque, yellowing, or deformity; smooth in surface; and intact at the edges	18.37%
Late Blight	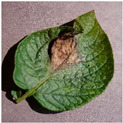	Potato late blight is characterized by irregular dark green or brown spots on the leaves, water stains on the edges of the spots, and white mold layers on the back of the leaves when wet	40.82%

**Table 2:** Experimental hardware and software parameters.

Hardware environment	CPU	12 vCPU Intel(R) Xeon(R) Platinum 8255C CPU
	GPU	RTX 3090
	RAM	375 GB
	Video Memory	24 GB
Software environment	OS	Ubuntu 20.04.4
	CUDA Toolkit	V11.3
	CUDNN	V8.2.4
	Python	3.8.10
	torch	1.11.0
	torchvision	0.14.1

**Table 3:** Convolutional comparison test.

Method	ACC	Precision	Recall	F1 Score
1 × 1 Convolution	0.8510	0.8496	0.8172	0.8252
Ghost Convolution	0.8612	0.8465	0.8406	0.8471
DSC	0.8529	0.8301	0.8183	0.8409
Atrous Convolution	0.8621	0.8547	0.8370	0.8431
Involution	0.8663	0.8318	0.8434	0.8290
CRMC	0.8714	0.8756	0.8522	0.8578

**Table 4:** Comparison of the effect of inserting different modules.

Method	ACC	Precision	Recall	F1 Score
Without attention	0.8510	0.8496	0.8172	0.8252
CBAM	0.8471	0.8455	0.8271	0.8124
CA	0.8557	0.8624	0.8257	0.8381
CCA	0.8634	0.8577	0.8319	0.8325
TA	0.8594	0.8412	0.8285	0.8352
STA	0.8755	0.8861	0.8469	0.8394

**Table 5:** Comparison of metrics for different optimizers.

Method	ACC	Precision	Recall	F1 Score
Adam	0.8602	0.8513	0.8206	0.8295
Lion	0.8696	0.8660	0.8283	0.8298
Radam	0.8688	0.8402	0.8474	0.8266
RMSprop	0.8471	0.8674	0.8120	0.8220
GWO	0.8422	0.8733	0.8422	0.8259
SSA	0.8445	0.8562	0.8694	0.8287
BLA	0.8745	0.8776	0.8537	0.8336

**Table 6:** Comparison of ablation results.

ResNext-50	**CRMC**	**STA**	**BLA**	**ACC**	**Precsion**
				0.8510	0.8496
	√			0.8714	0.8756
		√		0.8755	0.8861
			√	0.8745	0.8776
	√	√		0.8854	0.8991
	√		√	0.8984	0.8895
		√	√	0.8971	0.9039
	√	√	√	0.9204	0.9158

**Table 7:** Comparing the performance of CBSNet with other networks.

Methods	ACC	Precision	Recall	F1 Score
GoogLeNet [41]	0.8959	0.9299	0.8699	0.8806
EfficientNet V2 [42]	0.8694	0.8951	0.8831	0.8716
DensNet-121 [43]	0.8878	0.8941	0.8876	0.8860
MobileNetV3 [44]	0.8980	0.9146	0.8981	0.8974
ResNet-50 [45]	0.8571	0.8894	0.8446	0.8578
CMT [46]	0.9020	0.9095	0.9002	0.8972
CBSNet	0.9204	0.9158	0.9024	0.9071

**Table 8:** Generalization experiments conducted on public datasets.

Dataset	Method	ACC	Precision	Recall	F1 Score
Plant-Disease	Ours	0.9469	0.9474	0.9424	0.9466
	ResNext-50	0.9380	0.9349	0.9310	0.9457
CDICD	Ours	0.9714	0.9726	0.9583	0.9713
	ResNext-50	0.9677	0.9719	0.9777	0.9678

## Data Availability

If data are required, please contact the corresponding author at chenyd@usx.edu.cn.
